# International trends in primary liver cancer incidence from 1973 to 2007

**DOI:** 10.1186/s12885-015-1113-4

**Published:** 2015-03-04

**Authors:** Yue Zhang, Jian-Song Ren, Ju-Fang Shi, Ni Li, Yu-Ting Wang, Chunfeng Qu, Yawei Zhang, Min Dai

**Affiliations:** 1 National Office for Cancer Prevention and Control, Cancer Institute & Hospital, Chinese Academy of Medical Sciences/Peking Union Medical College, Beijing, 100021 China; 2 State Key Laboratory of Molecular Oncology, Cancer Institute & Hospital, Chinese Academy of Medical Sciences, Beijing, 100021 China; 3 Yale School of Public Health, Yale School of Medicine, Yale Cancer Center, New Haven, CT 06510 USA

**Keywords:** Liver neoplasms, Incidence, International trends, HBV, HCV

## Abstract

**Background:**

Primary liver cancer (PLC) is a common cancer worldwide, especially in developing countries. Several previous studies using different datasets have summarized PLC incidence rates and trends in different populations. However, with changes in exposure to risk factors and the implementation of preventive measures, the epidemiology of PLC worldwide may have changed.

**Methods:**

We extended the analyses using the latest data from Cancer Incidence in Five Continents over the 35-year period 1973–2007 from 24 populations in Americas, Asia, Europe and Oceania using Joinpoint regression analysis. We examined age-standardized rates (ASRs) of PLC by histologic subtypes for both males and females in 24 populations during the period 2003–2007.

**Results:**

We found that during the period 2003–2007, the highest ASRs for PLC were observed in some Asian populations, ranging from 19.0 to 26.7 per 100,000 in males and 4.8 to 8.7 per 100,000 in females. The international trends between 1973 and 2007 showed that ASRs for PLC were declining in several Asian populations. In contrast, ASRs for PLC were increasing in some European, American and Oceanian populations.

**Conclusions:**

Although the reasons were not fully clear for these trends, public health measures in Asian populations and HCV transmission in European, American and Oceanian populations were likely to have contributed to these patterns. Meanwhile, other possible risk factors such as the consumption of alcohol, obesity, and nonalcoholic fatty liver disease should also be concerned for the burden of PLC.

## Background

It was estimated that for the year 2012, primary liver cancer (PLC) incidence rates ranked fifth in men and ninth in women worldwide [1]. The number of incident cases of PLC was estimated to be 782,000 per year, including 554,000 in men and 228,000 in women [1]. PLC mortality rates ranked the second in both sexes in the world [1]. Five-year relative survival rate for USA tends to be 16.6% based on data from the Surveillance, Epidemiology, and End Results (SEER) (2004–2010) Program of the US National Cancer Institute [2]. In China, the age-standardized 5-year relative survival rate for liver cancer was 10.1% [3]. PLC is the major type of liver cancer, which is composed of several histologic subtypes, including hepatocellular carcinoma (HCC), cholangiocarcinoma (CC), and combined hepatocellular carcinoma and cholangiocarcinoma (cHCC-CC) [4].

Most of the PLC cases (85%) are diagnosed in developing countries. The highest incidence rates have been reported in the regions of Southeast Asia and sub-Saharan Africa [5]. In these high-incidence populations, except for Japan, chronic infection with hepatitis B virus (HBV) and aflatoxin exposure were recognized as major risk factors for PLC. In low-incidence populations, however, PLC was mainly associated to the chronic hepatitis C virus (HCV) infection. It was estimated that most HCC cases (approximately 80%) were associated with HBV and/or HCV infections [6]. Moreover, some recent studies indicated that alcohol-related liver diseases, smoking, immigration, and obesity were also possible risk factors linking to PLC [7-9].

Several previous studies [10-12] using different datasets had reported the international trends in PLC incidence rates, one of which [12] reported the global PLC incidence rates and trends for 1993–2002 (10-year period). However, with changes in exposure to risk factors and the implementation of protective measures, the epidemiology of PLC worldwide may have changed. To give a longer-term and more recent comprehensive picture on the current status of PLC worldwide, we extended the analyses for the 35-year period from 1973 to 2007 from 24 populations in Americas, Asia, Europe and Oceania. This data may provide more useful evidence for evaluating the effect of previous measures of PLC prevention and control, and may facilitate the development of future measures.

## Methods

### Incidence data

To examine the changing trends in the incidence of PLC over time, age-standardized (by Segi’s world standard population [13]) incidence rates (ASRs) by sex were obtained from Volumes 4–10 of Cancer Incidence in Five Continents (CI5) from the website of the International Agency for Research on Cancer (IARC) [14-20] in which all data is publicly available. Volumes 4–10 of CI5 generally provided data by 5-year periods: 1973–1977, 1978–1982, 1983–1987, 1988–1992, 1993–1997, 1998–2002 and 2003–2007. Incidence data from 2003 to 2007 by histologic subtypes (HCC, CC, other & unspecified carcinoma) were collected from 24 populations in four continents from Vol. 10 of CI5. Classification of PLC from Vols. 4, 5–8 and 9–10 of CI5 was coded according to the International Classification of Diseases (ICD) 8th (155), 9th (155) and 10th (C22) revisions, respectively.

Populations were chosen for inclusion in our study on the basis of the following criteria: (1) incidence for time periods at least as far back as 1983–1987; (2) an absence of changes in population coverage or of warnings regarding data quality reported in CI5 Vols. 4–10; and (3) a sufficiently large number of registered cases in CI5 Vol. 10 to enable analyses of recent rates by histologic subtypes (trends by histologic subtypes were not included in our study). Only one registry from each country was selected; if more than one registry met the basic criteria, the registry with the largest population was included in the analysis (expect for China which included Hong Kong and Shanghai). Twenty four populations were selected: four from the Americas (Canada, British Columbia [BC]; Colombia, Cali; USA, SEER: (9 registries: California: San Francisco; Connecticut; Georgia: Atlanta; Hawaii; Iowa; Michigan: Detroit; New Mexico; Utah; Washington: Seattle) Black/White), six from Asia (China, Hong Kong; China, Shanghai; India, Mumbai; Israel: Jews; Japan, Osaka Prefecture; Singapore: Chinese), five from Northern Europe (Denmark; Finland; Norway; Sweden; UK, England, North Western Region [NWR]), three from Western Europe (France, Bas-Rhin; Germany, Saarland; Switzerland, Geneva), four from elsewhere in Europe [21] (Southern and Central & Eastern Europe including Italy, Varese Province; Poland, Cracow; Slovakia; Spain, Navarra), and two from Oceania (Australia, New South Wales [NSW]; New Zealand). No African populations met all the inclusion criteria. However, four African populations (Algeria, Setif Wilaya; Egypt, Gharbiah; Uganda, Kyadondo; Zimbabwe, Harare: African) were chosen to describe the PLC incidence rates in the last time interval (2003–2007).

Incidence data for white and black populations in US were not included in CI5 vol. four (1973–1977) and vol. five (1978–1982), so we further referred to the US SEER dataset [22]. The SEER program is a population-based cancer registry system covering 18 registries and 28% of the US population. Long-term data from 1973 to 2010 were available from nine registries that included approximately 9.4% of the US population (based on 2010 census).

For New Zealand, we abstracted the data for 1983–1987 and 1988–2002 from CI5*plus* [23] which was part of CI5 databases and contained annual incidence data for a single registry or a group of populations in one country. The data for the last time period 2003–2007 were obtained from CI5 vol. 10.

### Data analysis

Incidence trends in ASRs of PLC were analyzed using Joinpoint regression (Joinpoint regression software, Version 3.5.3-May 2012, available through the Surveillance Research Program of the US National Cancer Institute). The permutation method was used for significance tests. Changes in annual incidence rates from PLC were calculated as annual percentage change (APC) in each segment. In the final model, the Joinpoint analysis provided average annual percentage change (AAPC). The significant test of APC and AAPC to 0 was also conducted.

Age-standardized incidence rates of PLC by histologic subtypes (HCC, CC and other & unspecified carcinoma) and sex for selected populations during the period 2003–2007 were integrated and calculated. Secular trends in ASRs were examined by registry and sex for every five-year period during 1973–2007. PLC trends from New Zealand were described during five-year periods from 1983–1987 to 2003–2007. Figures displaying the incidence trends were prepared using a semi-log scale to facilitate the comparison of temporal trends as well as magnitude. These data were plotted at the midpoint of each five-year interval.

## Results

ASRs for PLC in 2003–2007 were highest in some populations of Asia (China, Hong Kong; Japan; China, Shanghai; Singapore: Chinese) and Africa (Egypt and Zimbabwe), and much lower in most populations in Europe, Americas and Oceania (Tables 1 and 2). In Asian populations, ASRs for PLC were ranging from 19.0 to 26.7 per 100,000 in males and 4.8 to 8.7 per 100,000 in females, except for India and Israel (Jews) (5.2 and 3.1 per 100,000 in males, 2.4 and 1.4 per 100,000 in females, respectively). In most populations in Americas, Europe and Oceania, PLC incidence rates varied between 2.2-7.8 per 100,000 for males and 1.0-3.7 per 100,000 for females except for France (13.6 per 100,000 for males and 2.5 per 100,000 for females), Switzerland (13.1 per 100,000 for males and 3.0 per 100,000 for females), Italy (12.6 per 100,000 for males and 3.7 per 100,000 for females), and USA, Black population (11.6 per 100,000 for males and 3.1 per 100,000 for females).

**Table 1:** **International variation in primary liver cancer incidence rates for males, from 1973–1977 to 2003-2007**

Populations	Period of registry established	Mean of MV%^1^	1973-1977		2003-2007		Joinpoint analyses (1973-2007)				
			Cases	Rate^2^	Cases	Rate^2^	Trend 1 Period	Trend 2APC^3^(%)	Period	APC^3^(%)	AAPC^4^(%)
**Northern Europe**											
Denmark	1953-1957	88.0	424	2.9	947	4.1	1975-2005	0.8^#^			0.8^#^
			(1973-1976)								
Finland	1959-1961	88.3	473	3.2	1,192	5.2	1975-2005	1.2			1.2
			(1971-1976)								
Norway	1959-1961	85.7	230	1.6	421	2.2	1975-2005	0.8			0.8
Sweden	1959-1961	95.7	1,162	3.4	1,465	3.4	1975-2005	-0.7			-0.7
			(1971-1975)								
UK, England, NWR	1973-1977	44.7	157	1.1	1,250	4.4	1975-1985	6.0^#^	1985-2005	3.9^#^	4.6^#^
**Western Europe**											
France, Bas-Rhin	1975-1977	64.1	76	4.9	547	13.6	1975-1990	6.4^#^	1990-2005	0.5	3.4^#^
			(1975-1977)								
Germany, Saarland	1968-1972	64.5^a^	86	2.4	394	7.5	1975-1985	4.6	1985-2005	3.6^#^	3.9^#^
Switzerland, Geneva	1970-1972	70.3	100	9.7	214	13.1	1975-2005	0.9^#^			0.9^#^
**Europe, Other**											
Italy, Varese Province	1976-1977	55.5	64	6.9	516	12.6	1975-2005	2.6^#^			2.6^#^
			(1976-1977)								
Poland, Cracow	1965-1966	42.3	89	5.9	103	4.8	1975-2005	-1.5			-1.5
					(2003-2006)						
Slovakia	1973-1977	55.1	183	3.1	1,052	6.4	1975-2005	2.6			2.6
Spain, Navarra	1973-1977	51.2	8	0.5	217	7.8	1975-1985	28.9^#^	1985-2005	-1.8^#^	7.5^#^
**Americas**											
Canada, BC	1969-1972	56.5	142	2.0	201	5.2	1975-1995	3.7^#^	1995-2005	2.3	3.3^#^
Colombia, Cali	1967-1971	71.8	22	1.9	178	4.2	1975-2005	2.1^#^			2.1^#^
			(1972-1976)								
USA, SEER: Black	1973-1975	78.0^b^	154^c^	4.4	936	11.6	1975-1985	1.2	1985-2005	4.1^#^	3.1^#^
USA, SEER: White	1973-1975	74.7^b^	1,009^c^	2.1	4,443	5.9	1975-1985	1.4	1985-2005	4.6^#^	3.5^#^
**Oceania**											
Australia, NSW	1973-1977	73.8	139	1.1	1,379	5.4	1975-1995	6.5^#^	1995-2005	4.4	5.8^#^
New Zealand	1962-1966	61.1^d^	-	-	738	5.2	1985-2005	3.3^#^			3.3^#^
**Asia**											
China, Hong Kong	1974-1977	46.8	2,515	34.4	6,503	26.7	1975-2005	-0.8			-0.8
			(1974-1977)								
China, Shanghai	1975	14.2	949	31.7	6,128	21.7	1975-2005	-1.4^#^			-1.4^#^
			(1975)								
India, Mumbai	1964-1966	60.3	145	2.7	1,195	5.2	1975-2005	1.4			1.4
			(1973-1975)								
Israel: Jews	1960-1966	65.7	226	2.9	559	3.1	1975-2005	0.1			0.1
			(1972-1976)								
Japan, Osaka Prefecture	1966-1966	43.2	935	5.6	11,922	25.6	1975-1985	23.0^#^	1985-2005	-3.4	4.7^#^
Singapore: Chinese	1968-1972	32.3	843	32.2	1,451	19.0	1975-2005	-1.8^#^			-1.8^#^
**Africa**											
Algeria, Setif Wilaya	1986-1989	9.8	-	-	48	1.9	-	-	-	-	-
Egypt, Gharbiah	2003-2007	24.7	-	-	1,806	24.8	-	-	-	-	-
Uganda, Kyadondo	2003-2007	29.9	-	-	177	11.4	-	-	-	-	-
Zimbabwe, Harare: African	2003-2006	12.1	-	-	199	16.7	-	-	-	-	-
					(2003-2006)						

^1^Mean of MV% (Percentage of morphologically verified cases) was calculated from 1978 to 2007. ^2^Rate is age-standardized to the world population, per 100,000 person-years. ^3^APC, Annual Percent Change.^4^AAPC, Average Annual Percent Change. ^#^APC/AAPC is significantly different from 0 (two-side *p*<0.05).

^a^Germany, Saarland (1983-2007); ^b^USA, SEER: Black/White (1988-2007); ^c^The data of USA, SEER: Black/White were from SEER 9 registries database. ^d^New Zealand (1993-2007).

**Table 2:** **International variation in primary liver cancer incidence rates for females, from 1973–1977 to 2003-2007**

Populations	Period of registry established	Mean of MV%^1^	1973-1977		2003-2007		Joinpoint analyses (1973-2007)				
			Cases	Rate^2^	Cases	Rate^2^	Trend 1 Period	Trend 2APC^3^(%)	Period	APC^3^(%)	AAPC^4^(%)
**Northern Europe**											
Denmark	1953-1957	82.8	308	1.6	434	1.6	1975-2005	-0.4			-0.4
			(1973-1976)								
Finland	1959-1961	82.1	391	1.7	701	2.1	1975-2005	0.1			0.1
			(1971-1976)								
Norway	1959-1961	78.2	117	0.6	240	1.0	1975-2005	1.0			1.0
Sweden	1959-1961	94.3	754	1.8	859	1.7	1975-2005	-1.1			-1.1
			(1971-1975)								
UK, England, NWR	1973-1977	38.4	75	0.4	863	2.3	1975-2005	5.3^#^			5.3^#^
**Western Europe**											
France, Bas-Rhin	1975-1977	56.4	18	0.7	133	2.5	1975-2005	3.9^#^			3.9^#^
			(1975-1977)								
Germany, Saarland	1968-1972	54.4^a^	77	1.5	207	2.9	1975-2005	2.0^#^			2.0^#^
Switzerland, Geneva	1970-1972	66.9	23	1.3	66	3.0	1975-2005	2.4^#^			2.4^#^
**Europe, Other**											
Italy, Varese Province	1976-1977	48.9	37	2.7	224	3.7	1975-2005	1.0^#^			1.0^#^
			(1976-1977)								
Poland, Cracow	1965-1966	32.2	116	4.3	70	2.4	1975-2005	-3.1^#^			-3.1^#^
					(2003-2006)						
Slovakia	1973-1977	52.2	190	2.6	619	2.4	1975-2005	0.6			0.6
Spain, Navarra	1973-1977	38.1	11	0.6	89	2.2	1975-2005	1.4			1.4
**Americas**											
Canada, BC	1969-1972	52.6	87	1.1	97	1.7	1975-2005	1.2^#^			1.2^#^
Colombia, Cali	1967-1971	72.5	19	1.5	182	3.2	1975-1985	1.6	1985-2005	3.4^#^	2.8^#^
			(1972-1976)								
USA, SEER: Black	1973-1975	72.1^b^	68^c^	1.5	336	3.1	1975-1985	0.7	1985-2005	3.2^#^	2.4^#^
USA, SEER: White	1973-1975	71.0^b^	613^c^	1.0	1,762	1.9	1975-1985	0.7	1985-2005	3.0^#^	2.2^#^
**Oceania**											
Australia, NSW	1973-1977	71.7	56	0.4	634	1.9	1975-1990	3.6	1990-2005	7.7^#^	5.6^#^
New Zealand	1962-1966	55.2^d^	-	-	328	1.9	1985-2005	2.8^#^			2.8^#^
**Asia**											
China, Hong Kong	1974-1977	44.0	741	8.9	2,001	6.9	1975-2005	-0.6			-0.6
			(1974-1977)								
China, Shanghai	1975	13.1	278	9.1	2,419	7.1	1975-1985	1.5	1985-2005	-2.2^#^	-1.0^#^
			(1975)								
India, Mumbai	1964-1966	54.3	43	1.0	567	2.4	1975-1985	5.3^#^	1985-2005	0.4	2.0^#^
			(1973-1975)								
Israel: Jews	1960-1966	55.9	102	1.3	341	1.4	1975-2005	0.1			0.1
			(1972-1976)								
Japan, Osaka Prefecture	1966-1966	39.3	249	1.2	5,689	8.7	1975-1985	23.5^#^	1985-2005	-1.6	6.2^#^
Singapore: Chinese	1968-1972	30.1	231	7.1	471	4.8	1975-1985	-0.8	1985-2005	-1.9^#^	-1.5^#^
**Africa**											
Algeria, Setif Wilaya	1986-1989	92.5	-	-	40	1.6	-	-	-	-	-
Egypt, Gharbiah	2003-2007	24.2	-	-	433	6.2	-	-	-	-	-
Uganda, Kyadondo	2003-2007	29.0	-	-	131	8.7	-	-	-	-	-
Zimbabwe, Harare: African	2003-2006	13.9	-	-	101	13.9	-	-	-	-	-
					(2003-2006)						

^1^Mean of MV% (Percentage of morphologically verified cases) was calculated from 1978 to 2007. ^2^Rate is age-standardized to the world population, per 100,000 person-years. ^3^APC, Annual Percent Change.^4^AAPC, Average Annual Percent Change. ^#^APC/AAPC is significantly different from 0 (two-side *p*<0.05).

^a^Germany, Saarland (1983-2007); ^b^USA, SEER: Black/White (1988-2007); ^c^The data of USA, SEER: Black/White were from SEER 9 registries database. ^d^New Zealand (1993-2007).

**Figure 1:**
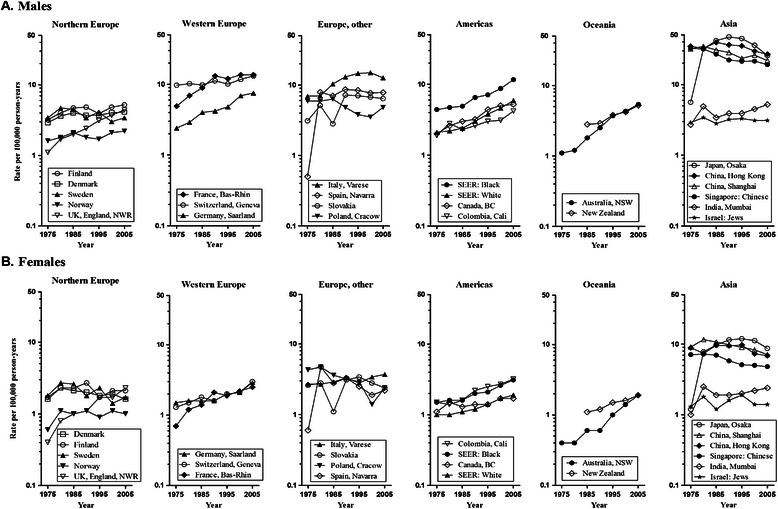
**Trends in age-standardized primary liver cancer incidence rates by continent and area for the time period 1973–2007: A. Males B. Females.**

Tables 1 and 2 also showed the results of Joinpoint analysis for ASRs in males and females for all ages, respectively. The secular trends in PLC incidence among 24 populations from 1973 to 2007 were presented in Figure 1. The increasing trends for both males and females in PLC incidence rates were seen in most of the populations in Europe, Americas, and Oceania. UK, England, France, Germany, Switzerland, Italy, Canada, Colombia, USA: Black, USA: White, Australia, and New Zealand (1982–2007) showed a significant increasing trend across all the periods (Tables 1 and 2 and Figure 1).

In males, ASRs for PLC in Germany, USA, Black, and USA, White increased significantly from the period 1982–1987 (Table 1 and Figure 1A). PLC incidence rates in France, Canada and Australia significantly increased from 1973–1977, leveled off in the 1990s (Table 1 and Figure 1A). ASRs for PLC in Spain significantly increased by 28.9% per year from 1973–1977, significantly decreased by 1.8% per year from 1982–1987, whereas ASRs for PLC in Finland, Norway, Sweden, Poland and Slovakia leveled off in all the period (Table 1 and Figure 1A). In females, the pattern of PLC incidence in each population seemed to be similar except for Denmark, Poland and Spain (Table 2 and Figure 1B). ASRs for PLC in Poland significantly decreased by 3.1% per year from 1973–1977 to 2003–2007, whereas ASRs for PLC in Denmark and Spain showed stable trends from 1973–1977 to 2003–2007.

However, in Asia, ASRs for PLC for both males and females showed significant decreasing trends in two of the six populations (China, Shanghai; Singapore: Chinese) from 1973–1977 to 2003–2007 (Tables 1 and 2 and Figure 1A and B). The stable trends among males and females were seen in two of the six populations (China, Hong Kong and Israel: Jews) from 1973–2007 to 2003–2007. ASRs for PLC in one of the six populations (Japan) significantly increased by 23.0% in males and 23.5% in females from 1973–1977 and reached a plateau in 1990s (Tables 1 and 2 and Figure 1A and B). Whereas ASRs for PLC for females in India significantly increased by 5.3% from 1973–1977 and leveled off in 1980s (Table 2 and Figure 1B).

**Figure 2:**
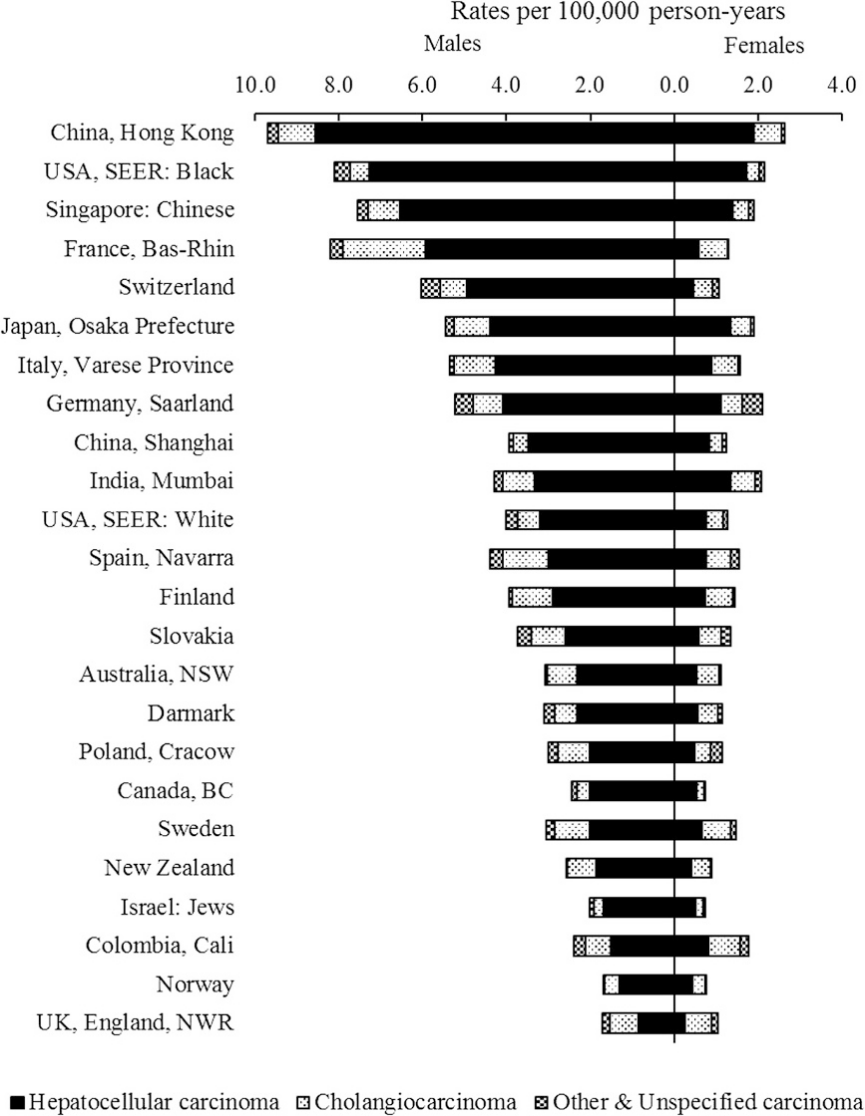
**Age-standardized primary liver cancer incidence rates by histologic subtypes for selected populations for the time period 2003–2007.**

According to the ASRs of PLC by histologic subtypes from 2003 to 2007, HCC was the leading histologic subtype, followed by CC and other & unspecified carcinoma (Figure 2). The highest incidence rate of HCC was observed in China, Hong Kong (8.5 per 100,000 in males and 1.9 per 100,000 in females), and the lowest one was shown in UK England (0.9 per 100,000 in males and 0.3 per 100,000 in females). The highest incidence of CC was seen in France (2.0 per 100,000 in males and 0.7 per 100,000 in females), followed by other European countries including Spain (1.1 per 100,000 in males and 0.6 per 100,000 in females), Finland (1.0 per 100,000 in males and 0.7 per 100,000 in females), and Italy (1.0 per 100,000 in males and 0.6 per 100,000 in females). China, Hong Kong (0.9 per 100,000 in males and 0.7 per 100,000 in females) and Japan (0.9 per 100,000 in males and 0.5 per 100,000 in females) had relatively higher incidence of CC than other Asian countries.

## Discussion

International trends in PLC incidence rates during the period 1973–2007 showed that the PLC incidence rates increased in most European, American and Oceanian populations, although these age-standardized PLC incidence rates in 2003–2007 were much lower than these in Asia. Meanwhile, PLC incidence rates decreased in Asian populations, although their age-standardized PLC incidence rates in 2003–2007 were the highest in the world.

PLC is a common cancer, particularly in Asia countries such as China, Japan and Singapore (Chinese). Among these countries, PLC is closely associated with hepatitis virus infection (HBV infection in China and Singapore, HCV infection in Japan) and exposure to aflatoxin (in China). In our study, the decreasing trends in China and Singapore may be attributed to some public health measures [24-27]. HBV vaccination was incorporated into the national childhood immunization program by China and Singapore from the middle 1980s to the early 1990s. The immunization coverage with three doses of HBV vaccine was 70.7%-95.0% in 1999 [28,29]. Several studies also reported the decreases in PLC incidence rates in China, particularly in Shanghai and in younger age groups [24,30]. Another study in Taiwan showed that the age- and sex- adjusted rate ratios for individuals aged 5 to 29 years decreased by more than 80% for HCC incidence from 1977–1980 to 2001–2004 [25]. In Singapore, Chia et al. [26] suggested that a general declining trend in liver cancer incidence was especially notable in local-born Chinese. Although the measure had no an effect on general population, we expect it will play an important role in the reduction of PLC incidence rates in the coming decades. Moreover, dietary aflatoxin exposure declined in the high-incidence areas of PLC seemed to have contributed to the decrease in PLC incidence in China [31]. A study in Qidong, China [31], where aflatoxin exposures were common, had reported that the decreasing liver cancer incidence in population over 25 years could mainly be attributable to the reduction of exposure to aflatoxin from 1980 to 2008.

In Japan, there were different trends between 1973–1992 and 1988–2007. The increasing trends started in 1973–1977 and reached peak in 1988–1992. This was thought to be in part due to the spread of HCV infection, which began in the 1920s and increase after World War II with an explosion in parenteral amphetamine use and paid blood donation [32,33]. Although APC did not significantly decrease during 1988–2007, the decline in PLC incidence had been continuously seen from 1988–1992 in our analysis. Stiffening of legal penalties against amphetamine use starting in 1954 and conversion from paid to voluntary blood donation in late 1960s might have reduced HCV transmission [34]. After the discovery of HCV RNA, HCV screening tests for first- and second-generation HCV antibodies started in 1989 and 1992, respectively [35]. These tests were adopted by the Red Cross Blood Center for screening blood, which further decreased the risk of post-transfusion hepatitis. The Japanese government has taken urgent comprehensive countermeasures against hepatitis (HBV and HCV) and HCC since 2002 [33]. Therefore, these measures would provide a significant contribution to decrease the number of patients suffering from HCV-related liver diseases including PLC.

In contrast, PLC was not a very common cancer in European, American and Oceanian countries where there were no epidemic regions of HBV infection. However, an increasing trend of PLC incidence rates was seen in most of these populations which were partly due to the widespread HCV infection associated with drug use, exposure to contaminated blood transfusion and/or needles used for medical purposes [36]. The natural history of PLC indicated that the time between exposure to HCV and development of HCC is about 30 years [37]. HCV infections were found in 30-50% of HCC patients in the United States and 44-66% of HCC patients in Italy [38]. Both of these countries had the highest PLC incidence rates in their own continent. The different peak years of HCV infection prevalence for each country were likely responsible for the respective peaks in PLC incidence rates. The increasing trends in PLC incidence rates in the United States could be attributed to increased HCV exposures by contact with contaminated blood and injection drug use during the 1960s and 1970s [39]. In Italy, the upsurge of liver cancer incidence after 1970s was in part attributable to HCV infection caused by the re-use of disposable syringes among intravenous drug users without proper disinfection [40]. Meanwhile, in several studies conducted in Western countries, 30 to 40% of patients with HCC did not have chronic infection with HBV or HCV, suggesting alternative causes [5]. Other factors including alcohol [41,42], obesity [43,44], and non-alcoholic fatty liver disease (NAFLD) [5] might be contributed to the increasing trend. In population-based cohort studies in the United States and Scandinavia [44-46], HCC was 1.5 to 2.0 times as likely to develop in obese persons as in those who were not obese. NAFLD, which is present in up to 90% of all obese persons and up to 70% of persons with type 2 diabetes, has been proposed as a possible risk factor for HCC [47]. Although there were still some difficult problems in the latency period from exposure to these factors and PLC development, more emphasis should be recommended to control these factors.

The advent of precise diagnostic tests may increase recognition of the disease, which accounts for a rising incidence, rather than a true increase in its occurrence [48]. Ultrasonography, measurement of serum alpha-fetoprotein, and computed tomography scanning have been routinely used since the early 1980s, which should lead to an increase in the number of hepatic biopsies conducted. However, the percentage of histologically confirmed PLC has not increased significantly during the study period in these countries which had an increasing trend in PLC incidence rates. In addition, females in Poland (from 1973–1977 to 2003–2007) and males in Spain (from 1982–1987 to 2003–2007) also exhibited a decreasing trend of PLC incidence rates. The reasons for this decreasing trend remain unclear.

This study has several strengths. The data were abstracted from large, well-established registries throughout the world. For the first time, data covering 35 years were analyzed to describe the variation of international trends in PLC incidence rates, which may stimulate further etiologic research. In addition, incidence rates for particular histologic subtypes of PLC in different populations were examined separately. One limitation of this study was that trends by histologic subtypes were not examined. The variation of ICD coding might influence the interpretation of our results. In our study, ICD coding contained ICD-8 (Malignant neoplasm of liver and intrahepatic bile ducts, specified as primary), ICD-9 (Malignant neoplasm of liver, specified as primary) and ICD-10 (Malignant neoplasm of liver and intrahepatic bile ducts, specified as primary). CC was not included in ICD-9 (period from 1978–1982 to 1993–1997). Therefore, the changes in PLC rates mainly reflect changes of HCC. Our study was also limited by the lack of nationwide cancer registries in some countries, thus registration data might not accurately reflect the true patterns in the respective countries.

## Conclusions

Our analysis on CI5 data suggested that ASRs for PLC were declining in several Asian countries where the highest incidence rates were still seen between 1973 to 1977 and 2003 to 2007. On the contrary, ASRs for PLC were increasing in some American, European and Oceanian countries. HBV vaccination programs and screening tests might play an important role in deceasing trends in Asia. Although the reasons of the increasing trends in American, European and Oceanian populations were not fully clear, the variation was likely to be due to in part the increasing prevalence of HCV infection. While a vaccine for HBV is widely available in most developed and developing countries, there is currently no vaccine available for HCV. Therefore, it is a critical for HCV infection prevention that blood donations are screened, safe injection practices are used at all times, and unnecessary injections are avoided. Additionally, controlling other risk factors such as alcohol consumption, obesity, and NAFLD may help to reduce PLC incidence rates.

## Abbreviations

PLC: Primary liver cancer
SEER: Surveillance, Epidemiology, and End Results
HCC: Hepatocellular carcinoma
CC: Cholangiocarcinoma
cHCC-CC: Combined hepatocellular carcinoma and cholangiocarcinoma
ASRs: Age-standardized incidence rates
HBV: Hepatitis B virus
HCV: Hepatitis C virus
CI5: Cancer Incidence in Five Continents
IARC: International Agency for Research on Cancer
ICD: International Classification of Diseases
BC: British Columbia
NWR: North Western Region
NSW: New South Wales
APC: Annual percentage change
AAPC: Average annual percentage change
HBsAg: Hepatitis B surface antigen
NAFLD: Nonalcoholic fatty liver disease
